# By-Passing Large Screening Experiments Using Sequencing as a Tool to Identify scFv Fragments Targeting Atherosclerotic Lesions in a Novel *In Vivo* Phage Display Selection

**DOI:** 10.3390/ijms13066902

**Published:** 2012-06-07

**Authors:** Kamel Deramchia, Marie-Josee Jacobin-Valat, Jeanny Laroche-Traineau, Stephane Bonetto, Stephane Sanchez, Pierre Dos Santos, Philippe Massot, Jean-Michel Franconi, Pierre Martineau, Gisele Clofent-Sanchez

**Affiliations:** 1 Magnetic Resonance Center of Biological Systems, UMR 5536, National Center for Scientific Research, Bordeaux Segalen University, 33076 Bordeaux Cedex, France; E-Mails: kamel@rmsb.u-bordeaux2.fr (K.D.); marie-josee.jacobin-valat@rmsb.u-bordeaux2.fr (M.-J.J.-V.); jeanny.laroche@rmsb.u-bordeaux2.fr (J.L.-T.); stephanebonetto@hotmail.com (S.B.); stephane.sanchez@rmsb.u-bordeaux2.fr (S.S.); philippe.massot@rmsb.u-bordeaux2.fr (P.M.); jean-michel.franconi@rmsb.u-bordeaux2.fr (J.-M.F.); 2 Technology Platform for Biomedical Innovation, Bordeaux Segalen University, 33600 Bordeaux Cedex, France; E-Mail: pierre.dossantos@wanadoo.fr; 3 MCRI, Montpellier Cancer Research Institute, INSERM, U896, Montpellier1 University, CRLC Val d’Aurelle Paul Lamarque, Montpellier, F-34298, France; E-Mail: pierre.martineau@inserm.fr

**Keywords:** atherosclerosis, human antibody, single-chain variable fragment (scFv), semi-synthetic phage display library, *in vivo* phage display, biopanning

## Abstract

Atherosclerosis is a chronic, progressive inflammatory disease that may develop into vulnerable lesions leading to thrombosis. To interrogate the molecular components involved in this process, single-chain variable fragments (scFvs) from a semi-synthetic human antibody library were selected on the lesions induced in a rabbit model of atherosclerosis after two rounds of *in vivo* phage display. Homing Phage-scFvs were isolated from (1) the injured endothelium, (2) the underlying lesional tissue and (3) the cells within the intima. Clones selected on the basis of their redundancy or the presence of key amino acids, as determined by comparing the distribution between the native and the selected libraries, were produced in soluble form, and seven scFvs were shown to specifically target the endothelial cell surface and inflamed intima-related regions of rabbit tissue sections by immunohistology approaches. The staining patterns differed depending on the scFv compartment of origin. This study demonstrates that large-scale scFv binding assays can be replaced by a sequence-based selection of best clones, paving the way for easier use of antibody libraries in *in vivo* biopanning experiments. Future investigations will be aimed at characterizing the scFv/target couples by mass spectrometry to set the stage for more accurate diagnostic of atherosclerosis and development of therapeutic strategies.

## 1. Introduction

Atherosclerosis is the main cause of death in the western world and incidence is increasing rapidly in developing countries. Atherosclerosis is regarded as an inflammatory disease that results from an initial activation of the endothelial cells with further enhancement of oxidative stress, lipid and leucocyte recruitment. The lesions may evolve into vulnerable plaques with large lipid cores covered by a thin fibrous cap that are at high risk of rupture and thrombi formation, thus precipitating the clinical conditions of stroke and myocardial infarction. Nowadays, there is an increasing interest in assessing the cellular components that underlie the risk of rupture for molecular imaging and therapeutic purposes. Even though progress in understanding the atherogenic molecular basis has been made, thus opening up horizons for several promising novel targets [1], a significant amount of research focuses on the discovery of new classes of agents able to target the atherogenic components [2–8]. The challenge has been given impetus by the rise in random screening technologies based on nanoparticle libraries [9,10], combinatorial chemistry [11–13] and phage display [14–17]. *In vivo* selection using phage-displayed peptide libraries has been particularly effective for identifying new target/ligand pairs specific to the vasculature of normal and diseased tissues [18–20]. Peptide sequences have been identified with homology to natural ligands, and these peptides recognize known or novel targets expressed on the activated endothelial cell surface [20].

Here we describe a novel procedure for selection of human antibody fragments (single-chain fragment variable (scFv)) that bind to diseased vasculature. Because of ethical considerations, human vasculature has not been widely used for *in vivo* phage selection approaches [21]. We thus developed a rabbit model for atherosclerosis. Rabbits were fed a high cholesterol diet and were subjected to two surgeries to induce an inflammatory process. We then performed two rounds of *in vivo* phage selection using a large semi-synthetic human single-chain antibody fragment library. This work differs from others by providing human antibodies as targeting agents. Antibodies exhibit advantages over other binding molecules including high specificities and affinities for their antigens and long half-lives *in vivo*. In addition, antibodies of human origin can be more easily translated into the clinic than rodent antibodies for safety reason. The human library we used is based on a unique framework with randomized CDR3 (complementary determining region 3) loops mimicking the natural human diversity. Phage-scFvs were selected that targeted the injured endothelium, the underlying lesional tissue and the cells within the intima. Clones were selected based on their redundancy or their amino acid distribution in the CDR3 loops [22]. ScFv fragments presenting amino acids over-represented at key positions in the VH or VL CDR3 are considered as being selected throughout the rounds of biopanning. This sequence-based approach allowed us to select the best clones without the large-scale screen usually required. The selected sequences were expressed as soluble scFv fragments, and seven of them were found to target rabbit atherosclerotic tissue sections by immunohistochemical approaches. These scFvs may constitute the basis for developing new clinical tools dedicated to molecular imaging and therapy.

## 2. Results and Discussion

### 2.1. Rabbit model of Atherosclerosis

**Figure 1:**
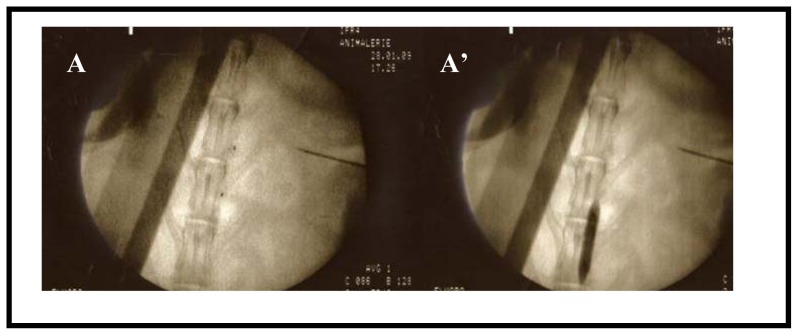
Inflammation induced in rabbits by hypercholesterolemic diet and surgeries. Rabbits were subjected to two surgeries: The mechanical removal of endothelial cells of the thoracic and abdominal aortic areas carried out by pulling back a Fogarty probe 4F along the endothelium; and, two months later, an angioplasty under radioscopic control with the aid of a balloon (20 mm long and 4.5 mm in diameter) inflated every 2 cm from the region of renal arteries to iliac bifurcation (**A**: balloon non inflated, **A’**: balloon inflated and pulled back).

**Figure 2:**
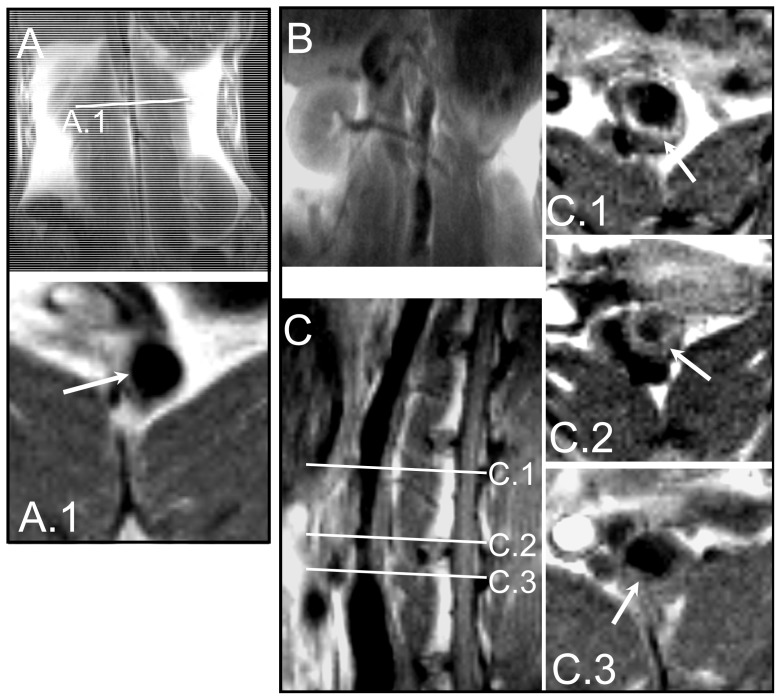
MRI of the abdominal aorta in control and atherosclerotic rabbits using a 0.2-T MRI system. Control rabbit coronal view (**A**) and enlarged image axial view (A.1) with position indicated by indicated by line A.1, showing normal vessel wall without plaque indicated by the white arrow. Atherosclerotic rabbit coronal view (**B**), sagittal views (**C**), and enlarged image axial views (C.1, C.2, C.3 corresponding respectively to positions indicated by lines C.1, C.2, C.3), showing thickened vessel wall and crescent-shaped plaque (white arrows).

To isolate phage-scFvs targeting atherosclerotic components of vulnerable plaques, we first developed a rabbit model of induced inflammation. In rabbits fed a high cholesterol diet, two kinds of surgery promoted the development of plaques reminiscent of human atherosclerotic lesions, in terms of cellular composition, patterns of lipid accumulation and growth characteristics [23] The second surgery, an angioplasty performed under radioscopic control, is illustrated in Figure 1. Before performing the *in vivo* phage display selection, it was crucial to know the state of development of the vulnerable plaques. To this end, we examined the atherosclerotic rabbit abdominal aorta with *in vivo* magnetic resonance imaging (MRI) and observed a thickened vessel wall and crescent-shaped plaques (Figure 2). The thickened intima, a hallmark of the pathology, is clearly visible on the sagittal slice (C). Arrows in panels C.1, C.2 and C.3, which correspond to axial views at positions indicated in panel C, depict a plaque that protrudes into the lumen of the vessel.

**Figure 3:**
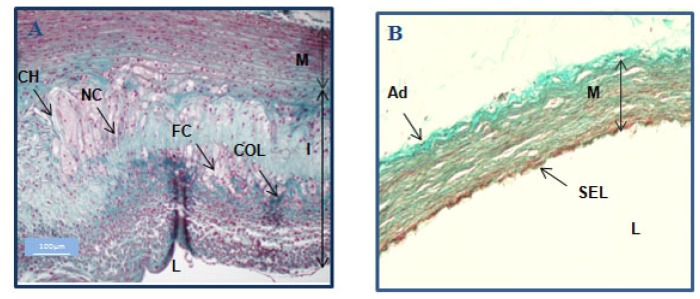
Histological analysis of control and atherosclerotic rabbits. Masson’s trichrome-stained histological slices illustrated the atherosclerotic plaques obtained in the vessel walls of a rabbit fed a high cholesterol diet and subjected to desendothelialization and angioplasty (**A**) and a control rabbit (normal diet, no surgery) (**B**). The aorta of the atherosclerotic animal showed complex plaque formation with intimal thickening and highly disorganized structures. Foam cells (FC) and collagen fibers (COL) (green) are interspersed. Necrotic cores (NC) and cholesterol (CH) were also present in the thick intima (I) of the atherosclerotic animal (**A**). In contrast, the control had a normal aorta with a single endothelial layer (SEL) covering a regular media (M) (**B**). L: lumen.

Histology proved that the protocol indeed induced highly vulnerable plaques with foam cells, necrotic cores and cholesterol. The intima is thickened and highly disorganized; macrophages and collagen fibers are interspersed as demonstrated by staining with Masson’s trichrome (Figure 3). Masson’s trichrome is a three-color staining protocol, well suited for distinguishing cells from surrounding connective tissue. Connective tissue is stained blue, nuclei are stained dark red/purple, and cytoplasm is stained red/pink. Foamy cells derived from macrophages as well as from smooth muscle cells (SMS) are the major cell types in atheroma. Foam cells, necrotic core and cholesterol crystals are easily distinguishable components of the vulnerable plaque, commonly described in the literature [24].

### 2.2. *In Vivo* Biopanning

The semi-synthetic scFv-fragment library was designed and constructed by Philibert *et al.* [22]. Briefly, the library is based on a unique framework of a human scFv, called scFv13R4. Diversity was introduced only in the CDR3 loops and eighteen sub-libraries were constructed as described in detail in the Experimental Section. Injection of the library was performed intravenously into the marginal ear vein of the rabbit. To ensure distribution of phage-scFvs in the tissues, we used an injection pump that provided constant flow and a circulation time optimized to 30 min [25]. These two factors combined allow both a saturation of non specific sites with phages not expressing scFv fragments (more than 90% of the total population) and a continuous supply of phage-scFvs, thus opposing clearance and catabolism via the major organs of the RES as reported by Zou and al [25]. Moreover, *in vivo* selection implies de facto that a negative selection occurs against the areas not affected by the disease. The animals were then sacrificed and the aortas extracted from the aortic arch to the iliac bifurcation.

In previous *in vivo* selections, performed in another model of atherosclerosis [26], we showed that the intravenous injection of phage-display scFv libraries permits the identification of candidates homing to atherosclerotic lesions not only in sites accessible to blood but also inside the lesions. Like tumors that have a vasculature much different from that of normal organs, atherosclerotic lesions have leaky endothelial cells. We thus hypothesized that phage-scFvs may directly contact sub-endothelial components and lesion-invading cells via the blood vessel or the vasa vasorum. We thus proceeded by a multi-step recovery and isolated phage-scFvs from three fractions in atherosclerotic tissue: the endothelium cell surface-bound phages-scFv (fraction F1), the intra-tissular fraction (F2) and the phages-scFv internalized in cells (F3). This is not a clear-cut recovery, which means that the intra-tissular fraction may contain tightly surface-bound phages-scFv and that the F3 fraction may be a sum of tightly bound, matrix-entrapped or real internalized phage-scFvs, probably captured in foam cells which constitute the main cellular components of the intima. This multi-step approach was performed around the idea of increasing phage-scFv recovery from the whole thickened intima, rich in targets over-expressed during the progression of the pathology. Most *in vivo* biopanning procedures were so far limited to the targets covering the vasculature surface.

**Figure 4:**
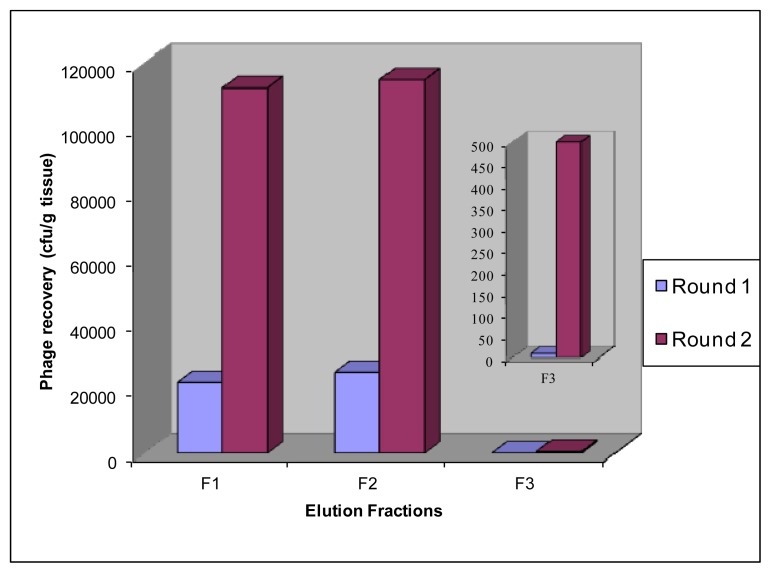
Enrichment of phages homing to atherosclerotic lesions by biopanning. Phage recovery was determined as colony forming units (cfu) per gram of tissue. Recovered phages were normalized for an input of 10^12^ phages. Phages were recovered from F1, F2 and F3 fractions (vascular surface, subjacent tissue and cellular compartment, respectively).

We performed only two rounds of *in vivo* biopanning in order to allow identification of *in vivo*-enriched binders without compromising diversity. It has been reported multiple selection rounds decreases the complexity of the selected repertoire [27]. At each round, we independently recovered F1, F2 and F3 fractions from diseased aortic tissue. From the second round of selection, we noted that the number of recovered phage-scFvs from affected tissue in F1, F2 and F3 fractions increased compared to the first round. The number of recovered phages after the second round of selection was 1.13 × 10^5^, 1.15 × 10^5^ and 500 cfu for fractions F1, F2 and F3 respectively (Figure 4). In other words, the number of output phage-scFvs normalized for an input of 10^12^ was increased by a factor 5 for the fractions F1 and F2, and by a factor 50 for the fraction F3. The fact that we not only show an enrichment in the fraction F1 but also in fractions F2 and F3, (with an even greater enrichment in the fraction F3), suggests to us that the implemented technology has led to the *in vivo* selection of scFvs able to reach subjacent lesional tissue layers and to be internalized within cells invading the intima.

### 2.3. Identification of *in Vivo*-Selected Clones by Sequencing

Two hundred colonies were randomly picked from the second round of biopanning from each fraction, selected by colony PCR to withdraw recombined plasmids harboring partial or complete deletions of the scFv gene and sequenced. Sequence analysis was performed using the IMGT/V-QUEST database. The VH and VL CDR3s were aligned and compared in length and amino acid distribution. We implemented a strategy to compare sequence homology between selected clones, taking into account the selection pressure inherent to the phage display technology. The rounds of selection should enrich for families of scFvs able to target biomarkers over-expressed in the pathology. Sequencing becomes valuable in situations where binding assays are challenging. Whereas sequencing information provides no direct information on the antigen recognized by the scFv, a set of homologous sequences is suggestive of binding events to related targets.

Before further binding validation, clones were thus selected according to the two following criteria. We first considered sequence redundancy; namely we required that VH CDR3, VL CDR3 or both variable CDR3 domains appeared at least twice in the pool of sequenced clones with a high identity score. To be assigned to the same family, we required less than 3 mutations per heavy or light chain domain. To determine this cutoff of 3 residues per CDR3, we counted the average number of residues of an antibody CDR3 loop that contacts the antigen. This was done by using the analysis of the antibody-protein contacts available in the AHo database [28]. In the VH [29] and VL [30] tables we counted the CDR3 residues that were buried by at least 60% upon antigen binding. In the case of VH CDR3, we obtained between 0 and 8 residues with a median of 3 and between 0 and 4 with a median of 2 for the VL CDR3. We thus used a unique cutoff of 3 residues for both chains.

Three sets of clones, C4.31.F2 (B2.32.F3), H8.32.F1 (I8.32.F1) and F8.32.F2 (E8.32.F2) were found twice in the selected library. In addition, A2.32.F3 and B4.31.F1, as well as A5.31.F1 and C5.31.F2, shared VH domains. Multiple occurrences of certain clones suggested a nonrandom accumulation and indicated an enrichment of lesion-specific clones.

We next considered a non library-encoded amino acid bias at a particular position as a sign of a specific scFv enrichment. None of the amino acid positions in CDR3 loops are truly random in natural human antibodies. The semi-synthetic library was designed to ensure a large functional diversity by mimicking both the length and the amino acid distribution of the CDR3 loops encountered in natural human antibodies. In addition, the diversity was focused in the two CDR3 loops. Philibert *et al.* validated this approach for the 5-amino acid long VH CDR3 and showed good overall agreement between the database of human antibody sequences, the library and the expected frequencies deduced from the oligonucleotide sequences [22]. We made the assumption that any selection pressure would lead to an over-incorporation of a particular amino acid at a given position, and we thus compared the frequencies of each amino acid at each position of the CDR3 sequences in the selected clones to those present in the library.

**Figure 5:**
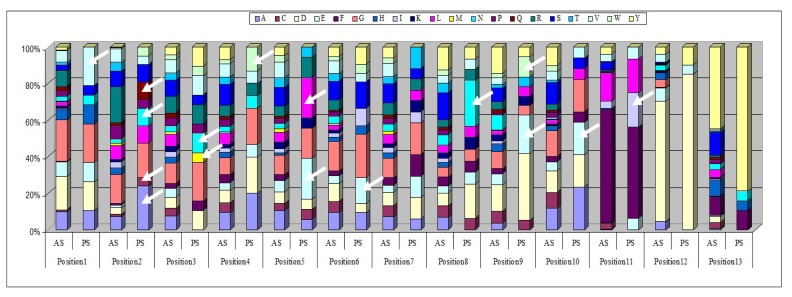
Amino acid distribution in 13 amino acid long VH CDR3 observed in sequenced clones at VH CDR3 loop. Distribution of amino acids at each position of the 13-amino acid long VH CDR3 from sequenced clones in the *in vivo* selected library (PS: post-selection) and predicted from the oligonucleotide sequences (T); see also additional file 2 in [22]. White arrows indicate over-represented amino acid after *in vivo* selection.

Randomly sequenced clones from the second round of biopanning were aligned by length, and the frequency of each of the 20 possible amino acids at each position for each loop length was calculated. We then compared this frequency with the expected distribution in the library, and the positions that diverged at least by a factor of 3 were highlighted. We illustrated our analysis in the case of the 13-amino acid long VH CDR3 (Figure 5). This figure highlights the identified positions and the biases in the amino acid distribution, for example A in position 2 and E in position 5. If the frequency of an amino acid at a given position was three fold higher after selection than the expected frequency, then we speculated that a “positive selection” had occurred. For each length, a “consensus” sequence (sequence with “hot” positions) was determined, taking into account over-expressed frequencies of some amino acid at a given position after *in vivo* selection compared to expected frequencies. Each clone was compared to this “consensus” sequence. We required that at least three residues be over-represented in the sequence to select the clone for further studies. This cutoff of 3 residues was chosen because it is the average number of contacts present in a CDR3 loop, as previously explained.

We first focused on the VH CDR3 domain since it is known that the heavy chain is more important for binding than the light chain [31]. Thirteen clones were selected from the most common loop lengths, 10, 13 and 14 amino acids (Table 1). The H8.32.F1 family was selected based on both criteria, redundancy and amino acid bias selection. The F8.32.F2 family, selected based on redundancy, was also included although only two positions were detected on the bias criterion. For other VH VDR3 lengths, only two residues were over-represented in some sequences or the low number of sequenced clones precluded relevant statistical analyses.

### 2.4. Expression of Selected Clones as Soluble scFv Fragments

**Figure 6:**
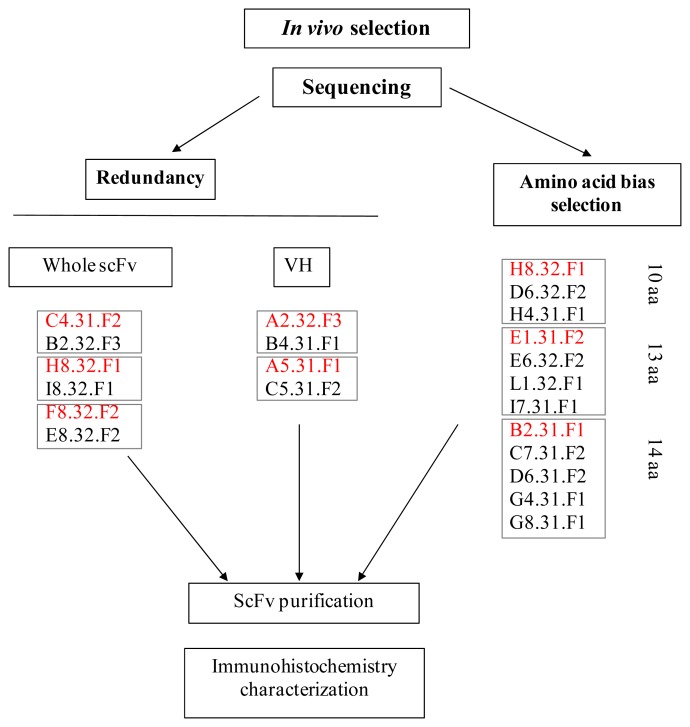
Scheme of the sequencing selection procedure. One clone (shown in red) in each selection set (redundancy in whole scFv or VH CDR3 domain or amino acid bias selection) was chosen for further characterization by immunohistochemistry.

**Figure 7:**
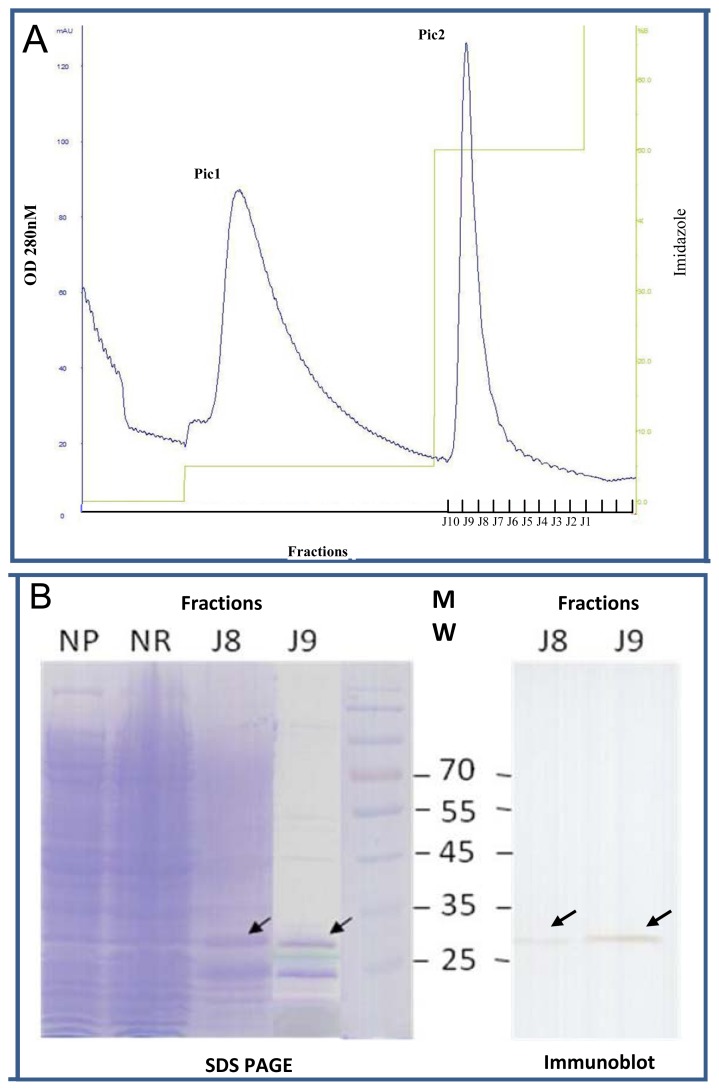
Profile of B2.31.F1 scFv purification by IMAC. The column was equilibrated with running buffer (20 mM Tris-HCl, pH 7.0). After sample loading, a wash with 25 mM imidazole (pH 7.0) removed non-specific contaminants (Pic1). ScFvs were eluted with 250 mM imidazole (pH 7.0) (Pic 2). Every recovered 1-mL fraction was evaluated for protein content by absorption at 280 nm (**A**) and then dialyzed against PBS for 16 h at 4 °C. Each fraction was then analyzed by SDS-PAGE (**B**).The presence of scFv in fractions J8 and J9 is indicated by an arrow. The fraction J9 presented higher purity than the J8 fraction. NP: Not purified lysate, NR: fraction not retained on the column. An immunoblot confirms the presence of scFv in fractions J8 and J9.

We chose one clone in each selection set for further characterization: C4.31.F2, H8.32.F1, F8.32.F2, A2.32.F3 and A5.31.F1 because they were found at least twice by homology in the selected library, and H8.32.F1, E1.31.F2 and B2.31.F1 for the 10, 13 and 14 amino acid classes in Table 1 (Figure 6). The selected clones were expressed as soluble scFv fragments using a non-suppressor *Escherichia coli* strain and were purified from bacterial lysates by immobilized metal affinity chromatography (IMAC) on a nickel affinity chromatography (Figure 7). After a wash with 25 mM imidazole to eliminate contaminants (Pic 1), scFvs were eluted with 250 mM imidazole (Figure 7A) (Pic 2). Figure 7B shows the SDS-PAGE profile of B2.31.F1 scFv purification and illustrates the high enrichment of soluble scFv preparation in the J9 fraction. The identity of the 30-kDa band was confirmed by immunoblotting using the 9E10 anti-c-myc monoclonal antibody as a probe.

### 2.5. Immunohistochemical Validation of scFv Human Antibody Fragments Selected by Sequencing

**Figure 8:**
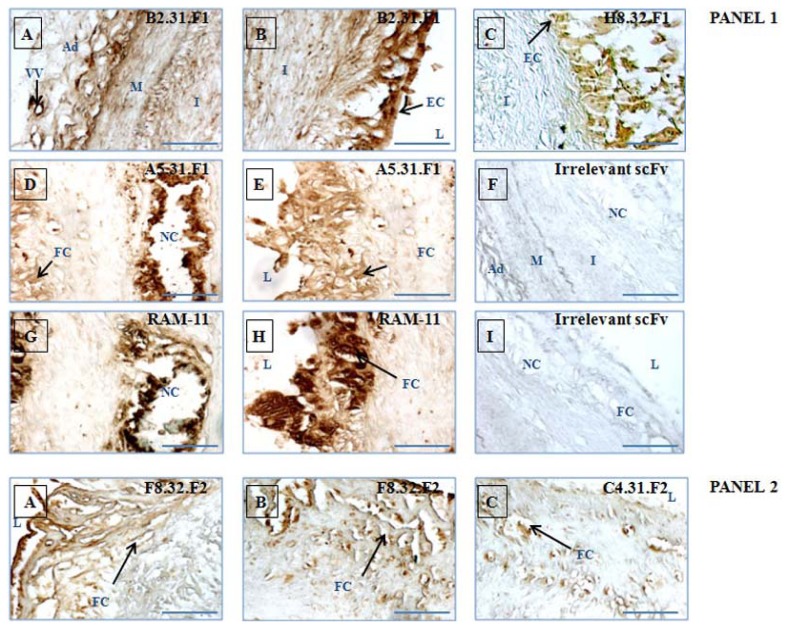

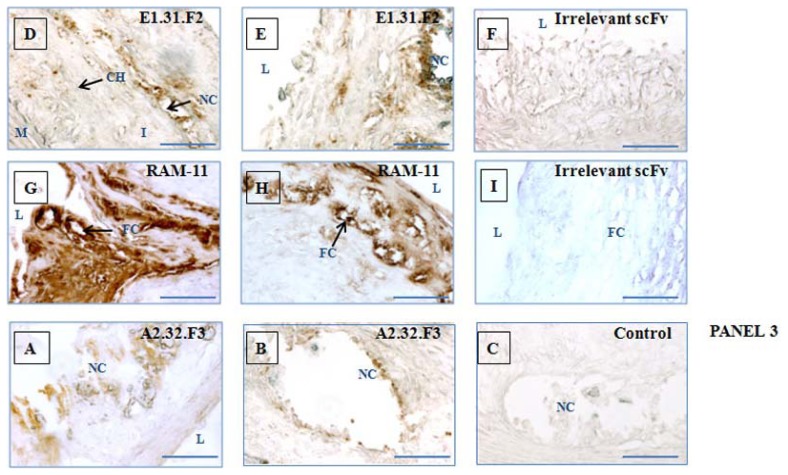
Immunodetection of scFvs in sections of vulnerable atherosclerotic lesions from atherosclerotic rabbits. Selected scFvs and irrelevant control scFv (random CDRH3 and CDRL3) were incubated with rabbit tissue sections. After addition of secondary antibodies, sections were treated with the DAB substrate kit reagent. The presence of the antigen recognized by scFvs was indicated by a yellow-brown stain. Incubation with B2.31.F1 (**panel 1**, **A**,**B**), H8.32.F1 (**panel 1**, **C**) and A5.31.F1 (**panel 1**, **D**,**E**) scFvs originating from the F1 fraction, F8.32.F2 (**panel 2**, **A**,**B**), C4.31.F2 (**panel 2**,**C**) and E1.31.F2 (**panel 2**, **D**,**E**) scFvs originating from the F2 fraction and A2.32.F3 scFv (**panel 3**, **A**,**B**) originating from the F3 fraction revealed the specific localization of each binding. Foam cell-rich advanced atherosclerotic lesions were stained with RAM-11 mAb (positive control) (**panel 1**, **G**,**H; panel 2**, **G**,**H**), whereas incubation with an irrelevant scFv produced no staining (**panel 1**, **F**,**I; panel 2**, **F**,**I**). No staining was observed in the presence of secondary antibody only (**panel 3**, **C**). L = artery lumen; I = intima; M = media; VV = vasa vasorum; NC = necrotic core; FC = foam cell; EC = endothelial cell; CH = cholesterol, Ad: Adventitia. Scale bars represent 100 μm.

Deparaffinized sections of diseased and healthy aorta were incubated with diluted scFv. Aliquots of 50 μL of scFv were stored directly after IMAC purification and dialysis at −80 °C and were thawed immediately before the experiment to avoid scFv degradation, thus contributing to excellent reproducibility of immunohistochemical results. For each slice, 10 μg/μL of scFv were used. Figure 8 shows staining (endothelium, lipid and necrotic core, fibrous area, cap) of a vulnerable plaque with selected human scFvs. The selected phage-scFvs, arising from different fractions of elution, were found to stain different areas of the intima, generally in accordance with the fraction they originated from. Staining by B2.31.F1 and H8.32.F1 isolated from F1 fraction was mainly in the endothelial and subendothelial layer and was weak in deeper intima (Figure 8, panel 1, A–C) whereas F8.32.F2, C4.31.F2 and E1.31.F2 isolated from the F2 fraction (Figure 8, panel 2, A–E) and A2.32.F3 isolated from the F3 fraction (Figure 8, panel 3, A,B) mainly stained the subjacent lesional intimal wall, some of them (E1.31.F2 (Figure 8, panel 2, D,E) and A2.32.F3 (Figure 8, panel 3, A,B)) staining components buried near necrotic cores. Two images of the same clone are provided when necessary to illustrate the labeling of different structures within the intimal thickening. For example, B2.31.F1 also strongly labeled the adventitia, highly irrigated by the vasa vasorum (indicated by a black arrow in panel 1 (A)). A5.31.F1 scFv, isolated from the F1 fraction, strongly labeled the lipid-rich necrotic area (Figure 8, panel 1, D), in addition to the targeting of the subendothelial layer (Figure 8, panel 1, E).

RAM-11 is a mouse antibody that reacts with a cytoplasmic antigen present in infiltrating rabbit macrophages. It thus recognizes macrophage-derived foam cells present immediately beneath the endothelium (Figure 8, panel 1, H) and more deeply within the intima, in a macrophage-rich area of atheromatous debris (Figure 8, panel 1, G) [32]. Using serial sections, we performed co-localization experiments with RAM-11 antibody for scFvs which target areas rich in macrophage-derived foam cells (Figure 8, panel 1, G,H; panel 2, G,H), The binding distribution of A5.31.F1 scFv, almost always found to be overlapped with RAM-11, (Figure 8, panel 1, D,E) suggests that the scFv may target a cell type, like macrophage, that migrates inside the vessel wall. The staining by two scFvs that were isolated from intra-tissular fraction (F2), F8.32.F2 and C4.31.F2, was most intense in the region beneath the endothelium rich in macrophage-derived foam cells, although diffuse staining was found throughout the intima (Figure 8, panel 2, A–C). The staining is not completely superimposable on that of RAM-11. The proteins targeted may be components of the sub-endothelial matrix. E1.31.F2 scFv also originated from the F2 fraction and showed a strong labeling in lipid-rich necrotic areas (Figure 8, panel 2, D,E). E1.31.F2 scFv and A2.32.F3 scFv (obtained from the F3 fraction (Figure 8, panel 3, A,B) exhibited a similar staining pattern in striking contrast to the distribution of the other scFvs. The epitopes recognized by E1.31.F2 and A2.32.F3 may be intra-tissular or cellular targets located in areas rich in lipids, atheromatous debris and in calcium deposits. However, even in the case of A5.31.F1 which clearly resembles staining obtained with RAM-11, it is impossible at this stage to assign a definitive cellular or extra-cellular location of the target recognized by these scFvs. The only way to attribute a target to an *in vivo* phage display isolated scFv is to perform immunoprecipitations of scFv/target pairs followed by mass spectrometry analyses.

No staining was obtained with an irrelevant scFv, randomly chosen from the unselected library, with all tissue sections (Figure 8, panel 1, F,I; panel 2, F,I). In addition, control sections with the secondary antibody only were devoid of any staining (Figure 8, panel 3, C). A similar staining procedure performed on vessel walls isolated from rabbits fed a normal diet yielded negative results with all antibody fragments (data not shown).

## 3. Experimental Section

### 3.1. Animal Model

All animal experiments were performed in accordance with the Guide for the Care and Use of Laboratory Animals (NIH Publication No. 85-23, revised 1996) and were approved by the local ethics committee. Adult male New Zealand rabbits (NZW), weighting 2.5 to 3.0 kg, were obtained from Charles Rivers Laboratories (St Germain sur l’Arbresle, France). Rabbits were fed a fat atherogenic diet including 0.3% cholesterol. To induce atherosclerosis, two surgeries were performed: The first surgery was performed two months after the beginning of the diet and the second was performed 2 months later to allow the formation of complex plaques with intramural thrombi.

In the first surgery, endothelial cells were removed with a specially designed Fogarty catheter (Fogarty 4F; Edwards Lifesciences), a catheter with an inflatable balloon near its tip. The Fogarty catheter was introduced over a guide wire with the tip placed at the level of diaphragm. The balloon was inflated and then pulled back to remove endothelial cells from the thoracic until the abdominal aorta. This process was repeated 3 times, and the catheter was removed. Two months later, the same rabbits were subjected to an angioplasty using an expandable latex balloon (Maxxum^®^, Boston Scientific; 20 mm long, diameter of 4.5 mm) that was firmly pressed against the arterial wall. The balloon for angioplasty was advanced through a femoral arteriotomy to the descendent thoracic aorta under radioscopic guidance. It was inflated every 2 cm from the region of renal arteries to iliac bifurcation.

During surgery, rabbits were anesthetized by the concurrent intramuscular injection of 20 mg/kg ketamine and 2 mg/kg xylazin. Anaesthesia was maintained with isoflurane gas (0.25% to 0.35%). As a preventive anti-thrombotic treatment, 1000 UI heparine (Héparine Choay^®^, Sanofi Synthélabo) was infused. Also administered as an analgesia was 100 mg aspirine (Aspégic^®^ injectable, Sanofi Synthélabo). A skin incision was made and the femoral artery was surgically exposed, the left for the removal of endothelial cells and the right for the angioplasty.

### 3.2. Magnetic Resonance Imaging

Rabbits were sedated using ketamine (25 mg/kg IM), xylazine (2 mg/kg IM) and butorphanol (0.12 mg/kg IM). Anesthesia was maintained during the MRI protocol with 2% isoflurane inhalation. Rabbits were positioned prone on the MRI table. Imaging was performed with a 0.2 T clinical MRI system (Siemens Open Viva, Erlangen, Germany). T1-axial images of the abdominal aorta were acquired with a spin-echo sequence including a black blood excitation pulse scheme with the following parameters: repetition time (TR): 400 ms, echo time (TE): 16 ms, section thickness: 5 mm, matrix: 128 × 128, field of view (FOV): 50 × 50 mm^2^, acquisition time: 20 min 32 sec, number of averages (NA): 12. T1-coronal and sagittal images of the abdominal aorta were obtained with a turbo spin echo sequence with the following parameters: TR: 450 ms, TE: 24 ms, section thickness: 6 mm, matrix: 246 × 256, FOV: 180 × 180 mm^2^, TA: 2 min 31 second NA: 2 (T1 coronal images) and TR: 450 ms, TE: 24 ms, section thickness: 4 mm, matrix: 246 × 256, FOV: 180 × 180 mm^2^, acquisition time: 7 min 26 second, NA: 6 (T1 sagittal images).

### 3.3. Human scFv Phage Library

A semi-synthetic scFv-fragment library was designed and constructed by Philibert *et al.* [22]. Briefly, the library is based on an unique framework of a human scFv, called scFv13R4, which is expressed at high levels in *Escherichia coli* cytoplasm. Diversity was introduced only in the CDR3 loops by using degenerate oligonucleotides so as to mimick the amino acid frequency at a given position along CDR3 lengths observed in natural human CDR3 loops. Eighteen sub-libraries were constructed: 13 VH sub-libraries with CDR3 loops ranging from 5 to 17 amino acids (encompassing most natural human CDR3H lengths) and 5 VL sub-libraries (2κ + 3λ) with CDR3 loops ranging from 9 to 10 amino acids for κ CDR3 and from 9 to 11 amino acids for λ CDR3. To remove non-functional scFvs, sub-libraries were screened for their ability to be expressed in frame with fusion protein CAT enzyme on selective CAM media before assembling in amounts proportional to the natural distribution of CDR3 loop lengths. The final library was constructed in the phagemid vector pCANTAB6 in fusion with the N-terminus of the minor pIII protein. Its diversity is about 1.5 × 10^9^ different variants.

### 3.4. *In Vivo* Biopanning in the Atherosclerotic New Zealand Rabbit Model

A continuous flow (170 μL/min) of PBS containing 1 × 10^13^ colony-forming units (cfu) of phagemid vector was injected into the marginal ear vein of rabbits for 30 min. The animals were then sacrificed and perfused via the heart with 120 mL of PBS to ensure phage clearance from the blood. The aorta was recovered from the aortic arch to the iliac bifurcation. The tissue was weighed, gently cut along the length and rinsed six times with 1 mL of cold PBS.

The endothelium cell surface-bound phages (fraction F1) were eluted with 950 μL of 0.1 M glycine-HCl, pH 2.2 for 8 min at room temperature under gentle agitation. Eluted phages were collected and immediately neutralized with 90 μL of 1 M Tris-HCl, pH 9.1 in a clean tube. The aorta was then washed three times with 500 μL of PBS (Ca^2+^, Mg^2+^ free). Washes were pooled and stored at 4 °C.

In order to elute intra-tissular phages (fraction F2), the aortic tissue was incubated with 900 μL of PBS (Ca^2+^, Mg^2+^ free) containing 2000 U/mL of collagenase type II (Gibco, France) adjusted to 1 mL with 2.5% Trypsin-EDTA (Eurobio, France) for 30 min at 37 °C with agitation. Dissociated tissue was homogenized with the aid of a Polytron homogenizer (Ultraturax TP-20, Kinematica, Lucerne, Swithzerland) on ice. The homogenate was centrifuged for 10 min at 5000× g to remove insoluble material. This one was homogenized again twice in the same way as tissue aorta. After centrifugation, supernatants containing eluted phages were collected, pooled in a clean tube with a protease-inhibitor cocktail added.

To access to internalized phages (fraction F3), the insoluble material was incubated with 500 μL of 0.1 M TEA (Sigma-Aldrich, France) for 5 min at room temperature with vigorous vortexing. Samples were neutralized by addition of 250 μL of 1M Tris-HCl, pH 7.4. After centrifugation at 1000× g for 10 min, the supernatant was collected.

Phage fractions F1, F2 and F3 were separately rescued by infection of XL1-blue *Escherichia coli* (Stratagene, La Jolla, CA, USA) grown to log phase in 25 mL of 2 × TY medium. After 45 min incubation infected bacteria were centrifuged and plated onto plates containing 2 × TY with ampicillin (100 μg/mL)/glucose (2% (w/v)) and incubated 16 h at 30 °C. The phage libraries were produced following the super-infection with M13KO7 helper phage (Invitrogen, Cergy-Pontoise, France) in 100 mL of 2 × TY containing ampicillin (100 μg/mL) and kanamycin (50 μg/mL) for 16 h at 25 °C. Phages were then purified from the supernatant culture by precipitation with 0.2 volumes of 20% (w/v) PEG 8000, 2.5 M NaCl. After centrifugation at 11,000× g for 45 min at 4 °C, the pellet containing phage particles was resuspended in a final volume of 500 μL sterile cold PBS and passed through a 0.22 μm pore-size filter. The titer of each production was around 3 × 10^13^ cfu/mL. One additional round of *in vivo* selection and amplification was performed with 1 × 10^12^ cfu.

### 3.5. Sequencing of Randomly Picked Clones

Bacterial colonies from the second round of biopanning were randomly picked for nucleotide sequence analysis. Sequencing was performed (MilleGen, Toulouse, France) using LMB3 primer (CAGGAAACAGCTATGAC) corresponding to the pCANTAB6 phagemid sequence downstream of the scFv insert. Sequence translation, comparison and alignment were done using IMGT/V-QUEST database [33].

### 3.6. Soluble scFv Production from Bacterial Culture

Selected scFv clones were expressed in HB2151 *Escherichia coli* (Amersham Pharmacia Biotech, Uppsala, Sweden) using a previously described procedure [25]. Briefly, selected XL1-blue *Escherichia coli* clones were super-infected with M13KO7 helper phage and supernatant containing phages was used to infect 20 μL of HB2151 *Escherichia coli* host (Amersham Pharmacia Biotech, Uppsala, Sweden) in log growth phase. After 45 min and centrifugation, infected bacteria were plated onto plates containing 2 × TY with ampicillin (100 μg/mL) and glucose (2% (w/v)) and incubated for 16 h at 30 °C. HB2151 clones were then cultured in 2 × TY containing ampicillin (100 μg/mL) and glucose (2% (w/v)) until log growth was reached. After spinning, the scFv production was induced by addition of 50 mL of 2 × TY containing ampicillin (100 μg/mL), glucose (0.1% (w/v)) and IPTG (200 mM) for 16 h at 25 °C.

### 3.7. scFvs Purification on Nickel-Affinity Chromatography

HB2151 cells were lysed as previously described using ice-cold osmotic choc buffer (1M Tris-HCl, pH 8, 1 mg/mL lysozyme, 1 mM PMSF, protease inhibitors cocktail (Roche Diagnostics) for 1 h at 4 °C on rotary shaker and 2 cycles of sonication. After centrifugation at 20,000× g for 30 min at 4 °C to remove insoluble material, 6His-tagged scFvs contained in the supernatant were purified by IMAC using Ni-NTA resin (Pierce, Bezons, France) using a Biopilot Chromatographic System (Amersham Biosciences, Sacley, France).

### 3.8. Histological Examination

Aortic specimens were embedded in paraffin and sectioned at 10 μm. The sections were deparaffinized and stained using a standard Masson-Trichrome protocol for morphological evaluation by light microscopy. Magnification of atherosclerotic plaque is ×100; magnification of normal vessel wall is ×200. Scale bars represent 100 μm.

### 3.9. Immunohistochemical Analysis of Rabbit Sections

Paraffin-embedded sections were prepared from arterial tissue. The 10-μm thick sections were deparaffinized and re-hydrated before neutralizing endogenous peroxidase activity with 3% H_2_O_2_/H_2_O for 5 min. Non-specific binding was blocked with 4% (w/v) BSA in PBS solution for 30 min at room temperature. Nickel-purified scFv fractions were diluted in 4% BSA in PBS (final concentration scFv between 5 and 10 μg/mL) and incubated 16 h at 4 °C. Following a wash with PBS, a mix of 1:250 anti-c-myc (Miltenyi Biotec, Paris, France) and 1:250 anti-6His (Roche) mAbs in 4% BSA/PBS were applied to the sections for 3 h at room temperature. After washing, the sections were incubated with 1:500 diluted HRP-conjugated anti-mouse IgG (Immunotech, Marseille, France) for 1 h at room temperature. Bound secondary antibody was detected with the DAB substrate kit (Vector Laboratories, Peterborough, UK) yielding a yellow brown color upon reaction. After washing, the sections were dehydrated and mounted in DPX medium.

## 4. Conclusions

We used a novel rabbit model of atherosclerosis and an *in vivo* phage display biopanning procedure to directly interrogate the molecular repertoire of diseased tissue in living animals. We selected scFv antibodies for validation by comparative sequencing, thus by-passing large screening experiments. Altogether the presence of redundant sequences of several clones and multiple “over-incorporation” of amino acids in a particular position indicates an enrichment of clones following the second round of *in vivo* biopanning. The relevance of the approach was further confirmed by immunostaining which is considered as being the most accurate *in vitro* study to keep the targets in their native environment. In future experiments, we will determine whether clones isolated in the same set (Figure 6) recognize the same or a closely related antigen. The strategy of selection by comparative sequencing implemented in this preliminary study will then be applied to other randomly picked colonies in order to increase the number of sequenced clones in VH CDR3 lengths other than 10, 13 and 14 CDR3 amino acids and in all the VL CDR3s. The selection of clones according to their “over-incorporation” of amino acids in particular position has similarities with the appearance of somatic mutations in the maturation of antibody affinity in the humoral immune response. It may be that the amino acid identified as conserved at a given position for a given CDR3 length will prove critical for the antibody affinity.

As reported in Pluckthun’s work, based on the calculated antigen-antibody contacts [29], we considered that there are on average two or three strong contacts per CDR3 in antigen-antibody recognition areas.

The *in vivo* use of phage-display libraries has also been reported in cancer patients to identify tumor-targeting ligands [21]. *In vivo* selection, through the wide panel of markers targeted on vasculature, allows identification of many target/ligand pairs [21,34,35]. However, most of the *in vivo* procedures use random phage libraries expressing peptides on their surface [17,19,20]. In spite of a few reported works [26,36–38], debate persists on the difficulties of using antibody fragment libraries *in vivo* selection strategy. One reason for use of peptide libraries is ease of alignment of short sequences of amino acids to find consensus motifs. Our study highlights the feasibility of selection of scFv fragments by amino acid distribution comparison before and after *in vivo* selection. Human antibodies and human antibody fragments such as scFvs exhibit many advantages over peptides including high specificity and high affinity binding.

The advantages of *in vivo* biopanning relative to cell-based screens include the presence of the complete panel of relevant targets not necessarily available *in vitro*, targets in their native conformation, identification of ligands that are able to reach their targets *in vivo* and the possibility of identifying novel biomarkers of relevance for *in vivo* targeting. By targeting intra-tissular molecules, our approach to a complex atherosclerotic mammalian model expands the field of the *in vivo* phage display selection applications, which may be extended to other disorders and especially to those with a strong angiogenic component.

Having at our disposal a large panel of different human scFv antibody fragments could open up avenues (1) for the discovery of new sets of relevant targets by immunoprecipitation with purified scFv and mass spectrometry antigen identification [26,36] and (2) for combining human antibodies recognizing different biomarkers for targeted imaging and therapy. One of our priorities in future studies will be to use these antibodies to functionalize nano-cargoes for Magnetic Resonance Imaging-guided therapy of the disease. The use of fully human antibodies is mandatory for clinical applications. This is why we developed *in vivo* screening strategies of human antibody libraries on animal models of the human disease. We reasoned that selecting phage scFvs *in situ* will give us the best chance to focus on ligands able to target overexpressed and accessible molecules in the context of the pathology. Selected scFvs will be linked to nano-cargoes loaded with contrast agents, enabling us to follow localization of our ligands using a Magnetic Resonance Imaging approach. Homing of the targeted contrast agent to atherosclerotic lesions will be controlled in cholesterol-fed ApoE mice on a 4.7 T superconducting magnet using 3D gradient echo sequences. Their biodistribution across tissues will be assessed by electron spin resonance (ESR) spectroscopy [39]. In a recent paper, we reported an *in vivo* MRI assay for visualizing P-selectin target with a specific MR contrast agent, using a murine anti-P-selectin antibody [40]. This proof-of-concept will form the basis for further development of MR nano-objects functionalized with human antibodies that could be directly translated into human clinical use.

## Figures and Tables

**Table 1:** Selection of clones by comparison of VH CDR3 amino acid distribution. VH CDR3 sequences predicted from the native library and VH CDR3 sequences from the selected library were compared at each amino acid position. A “consensus” (sequence with “hot” positions) was determined on the basis of over-expressed amino acids in sequenced clones from the second round of *in vivo* selection at a given position of the CDR3 length. Each amino acid position is numbered. Amino acids reported in the “consensus” are those whose frequency is significantly higher (≥3) than that of the initial library. X indicates that, at a given position, the amino acid distributions were equivalent in the library and after selection. Clones with 10, 13 and 14 amino acid long VH CDR3 loops are aligned with the “consensus” sequence. Identity with an over-expressed amino acid is highlighted.

**VH CDR3 Length: 10**																	
POSITION *				1	2	3	4	5	6	7	8	9	10				
CONSENSUS **		V	R	X	X	D/I	R	X	Y	X	V	E	D				
CLONES	F8.32.F2	V	R	X	X	I	X	X	X	X	X	E	X				
	H8.32.F1	V	R	X	X	D	X	X	X	X	V	X	D				
	D6.32.F2	V	R	X	X	X	R	X	Y	X	X	E	X				
	H4.32.F1	V	R	X	X	D	X	X	Y	X	V	X	X				
**VH CDR3 Length: 13**																	
POSITION *				1	2	3	4	5	6	7	8	9	10	11	12	13	
CONSENSUS **		V	R	V	A/C/N/Q	M/N	W	E/L	E	X	N	W/E	E	E	X	X	
CLONES	E1.31.F2	V	R	X	A	X	X	E	X	X	N	X	X	X	X	X	
	E6.32F2	V	R	X	N	X	X	L	X	X	N	X	X	X	X	X	
	L1.32F1	V	R	V	N	N	X	X	X	X	N	E	X	X	X	X	
	I7.31.F1	V	R	X	X	N	W	X	X	X	X	X	E	X	X	X	
**VH CDR3 Length: 14**																	
POSITION *				1	2	3	4	5	6	7	8	9	10	11	12	13	14
CONSENSUS**		V	R	E/S	D/E/M	W	I/R/W	P	W/M	G	X	E	X	X	Y	X	N
CLONES	B2.32.F1	V	R	E	X	X	W	P	X	X	X	E	X	X	X	X	X
	C7.31CF2	V	R	S	M	X	I	X	X	X	X	X	X	X	Y	X	X
	D6.31.F2	V	R	E	E	X	W	X	X	X	X	X	X	X	X	X	X
	G4.31.F1	V	R	X	D	X	R	X	M	X	X	X	X	X	X	X	N
	G8.31.F1	V	R	E	X	W	X	X	W	G	X	X	X	X	X	X	X

* Position of the amino acid in the CDR3 length just after V and R common amino acids;

** “consensus” sequence of over-represented amino acids established after analysis of amino acid distribution after *in vivo* selection.

## References

[1] Blanco-Colio L.M., Martin-Ventura J.L., Vivanco F., Michel J.B., Meilhac O., Egido J. (2006). Biology of atherosclerotic plaques: What we are learning from proteomic analysis. Cardiovasc. Res.

[2] Wickline S.A., Neubauer A.M., Winter P.M., Caruthers S.D., Lanza G.M. (2007). Molecular imaging and therapy of atherosclerosis with targeted nanoparticles. J. Magn. Reson. Imaging.

[3] Shaw S.Y. (2009). Molecular imaging in cardiovascular disease: Targets and opportunities. Nat. Rev. Cardiol.

[4] Antoniades C., Psarros C., Tousoulis D., Bakogiannis C., Shirodaria C., Stefanadis C. (2010). Nanoparticles: A promising therapeutic approach in atherosclerosis. Curr. Drug Deliv.

[5] Koenig W., Khuseyinova N. (2007). Biomarkers of atherosclerotic plaque instability and rupture. Arterioscler. Thromb. Vasc. Biol.

[6] Briley-Saebo K.C., Mulder W.J., Mani V., Hyafil F., Amirbekian V., Aguinaldo J.G., Fisher E.A., Fayad Z.A. (2007). Magnetic resonance imaging of vulnerable atherosclerotic plaques: Current imaging strategies and molecular imaging probes. J. Magn. Reson. Imaging.

[7] Rader D.J., Daugherty A. (2008). Translating molecular discoveries into new therapies for atherosclerosis. Nature.

[8] Sanz J., Fayad Z.A. (2008). Imaging of atherosclerotic cardiovascular disease. Nature.

[9] Weissleder R., Kelly K., Sun E.Y., Shtatland T., Josephson L. (2005). Cell-specific targeting of nanoparticles by multivalent attachment of small molecules. Nat. Biotechnol.

[10] Lam K.S., Salmon S.E., Hersh E.M., Hruby V.J., Kazmierski W.M., Knapp R.J. (1991). A new type of synthetic peptide library for identifying ligand-binding activity. Nature.

[11] Alvim J., Severino R.P., Marques E.F., Martinelli A.M., Vieira P.C., Fernandes J.B., da Silva M.F., Correa A.G. (2010). Solution phase synthesis of a combinatorial library of chalcones and flavones as potent cathepsin V inhibitors. J. Comb. Chem..

[12] Aina O.H., Liu R., Sutcliffe J.L., Marik J., Pan C.X., Lam K.S. (2007). From combinatorial chemistry to cancer-targeting peptides. Mol. Pharm.

[13] Toepert F., Knaute T., Guffler S., Pires J.R., Matzdorf T., Oschkinat H., Schneider-Mergener J. (2003). Combining SPOT synthesis and native peptide ligation to create large arrays of WW protein domains. Angew. Chem. Int. Ed. Engl.

[14] Iwamoto S., Nishimichi N., Tateishi Y., Sato Y., Horiuchi H., Furusawa S., Sawamura T., Matsuda H. (2009). Generation and characterization of chicken monoclonal antibodies against human LOX-1. MAbs.

[15] Hong H.Y., Lee H.Y., Kwak W., Yoo J., Na M.H., So I.S., Kwon T.H., Park H.S., Huh S., Oh G.T. (2008). Phage display selection of peptides that home to atherosclerotic plaques: IL-4 receptor as a candidate target in atherosclerosis. J. Cell. Mol. Med.

[16] Burtea C., Laurent S., Port M., Lancelot E., Ballet S., Rousseaux O., Toubeau G., Vander Elst L., Corot C., Muller R.N. (2009). Magnetic resonance molecular imaging of vascular cell adhesion molecule-1 expression in inflammatory lesions using a peptide-vectorized paramagnetic imaging probe. J. Med. Chem.

[17] Yao V.J., Ozawa M.G., Trepel M., Arap W., McDonald D.M., Pasqualini R. (2005). Targeting pancreatic islets with phage display assisted by laser pressure catapult microdissection. Am. J. Pathol.

[18] Kelly K.A., Nahrendorf M., Yu A.M., Reynolds F., Weissleder R. (2006). *In vivo* phage display selection yields atherosclerotic plaque targeted peptides for imaging. Mol. Imaging Biol.

[19] Liu C., Bhattacharjee G., Boisvert W., Dilley R., Edgington T. (2003). *In vivo* interrogation of the molecular display of atherosclerotic lesion surfaces. Am. J. Pathol.

[20] Houston P., Goodman J., Lewis A., Campbell C.J., Braddock M. (2001). Homing markers for atherosclerosis: Applications for drug delivery, gene delivery and vascular imaging. FEBS Lett.

[21] Krag D.N., Shukla G.S., Shen G.P., Pero S., Ashikaga T., Fuller S., Weaver D.L., Burdette-Radoux S., Thomas C. (2006). Selection of tumor-binding ligands in cancer patients with phage display libraries. Cancer Res.

[22] Philibert P., Stoessel A., Wang W., Sibler A.P., Bec N., Larroque C., Saven J.G., Courtete J., Weiss E., Martineau P. (2007). A focused antibody library for selecting scFvs expressed at high levels in the cytoplasm. BMC Biotechnol.

[23] Rekhter M.D., Hicks G.W., Brammer D.W., Work C.W., Kim J.S., Gordon D., Keiser J.A., Ryan M.J. (1998). Animal model that mimics atherosclerotic plaque rupture. Circ. Res.

[24] Phinikaridou A., Hallock K.J., Qiao Y., Hamilton J.A. (2009). A robust rabbit model of human atherosclerosis and atherothrombosis. J. Lipid Res.

[25] Zou J., Dickerson M.T., Owen N.K., Landon L.A., Deutscher S.L. (2004). Biodistribution of filamentous phage peptide libraries in mice. Mol. Biol. Rep.

[26] Deramchia K., Jacobin-Valat M.J., Vallet A., Bazin H., Santarelli X., Sanchez S., Dos Santos P., Franconi J.M., Claverol S., Bonetto S., Clofent-Sanchez G. (2012). New human antibody fragments homing to atherosclerotic endothelial and subendothelial tissues: An *in vivo* phage display targeting human antibodies homing to atherosclerotic tissues. Am. J. Pathol.

[27] De Wildt R.M., Mundy C.R., Gorick B.D., Tomlinson I.M. (2000). Antibody arrays for high-throughput screening of antibody-antigen interactions. Nat. Biotechnol.

[28] Honegger A., Pluckthun A. (2001). Yet another numbering scheme for immunoglobulin variable domains: An automatic modeling and analysis tool. J. Mol. Biol.

[29] AHo’s Amazing Atlas of Antibody Anatomy.

[30] AHo’s Amazing Atlas of Antibody Anatomy.

[31] Wilson I.A., Stanfield R.L. (1994). Antibody-antigen interactions: New structures and new conformational changes. Curr. Opin. Struct. Biol.

[32] Tsukada T., Rosenfeld M., Ross R., Gown A.M. (1986). Immunocytochemical analysis of cellular components in atherosclerotic lesions. Use of monoclonal antibodies with the Watanabe and fat-fed rabbit. Arteriosclerosis.

[33] IMGT/V-QUEST, IMGT/V-QUEST Programme, version 3.2.25.

[34] Arap W., Kolonin M.G., Trepel M., Lahdenranta J., Cardo-Vila M., Giordano R.J., Mintz P.J., Ardelt P.U., Yao V.J., Vidal C.I. (2002). Steps toward mapping the human vasculature by phage display. Nat. Med.

[35] Rajotte D., Arap W., Hagedorn M., Koivunen E., Pasqualini R., Ruoslahti E. (1998). Molecular heterogeneity of the vascular endothelium revealed by *in vivo* phage display. J. Clin. Invest.

[36] Robert R., Jacobin-Valat M.J., Daret D., Miraux S., Nurden A.T., Franconi J.M., Clofent-Sanchez G. (2006). Identification of human scFvs targeting atherosclerotic lesions: Selection by single round *in vivo* phage display. J. Biol. Chem.

[37] Johns M., George A.J., Ritter M.A. (2000). *In vivo* selection of sFv from phage display libraries. J. Immunol. Methods.

[38] Ueberberg S., Meier J.J., Waengler C., Schechinger W., Dietrich J.W., Tannapfel A., Schmitz I., Schirrmacher R., Koller M., Klein H.H. (2009). Generation of novel single-chain antibodies by phage-display technology to direct imaging agents highly selective to pancreatic beta- or alpha-cells *in vivo*. Diabetes.

[39] Chertok B., Cole A.J., David A.E., Yang V.C. (2010). Comparison of electron spin resonance spectroscopy and inductively-coupled plasma optical emission spectroscopy for biodistribution analysis of iron-oxide nanoparticles. Mol. Pharm.

[40] Jacobin-Valat M.J., Deramchia K., Mornet S., Hagemeyer C.E., Bonetto S., Robert R., Biran M., Massot P., Miraux S., Sanchez S. (2010). MRI of inducible P-selectin expression in human activated platelets involved in the early stages of atherosclerosis. NMR Biomed.

